# Water-air CO_2_ fluxes in the Tagus estuary plume (Portugal) during two distinct winter episodes

**DOI:** 10.1186/s13021-014-0012-3

**Published:** 2015-01-16

**Authors:** Ana P Oliveira, Marcos D Mateus, Graça Cabeçadas, Ramiro Neves

**Affiliations:** 1 Instituto Português do Mar e da Atmosfera (IPMA), I.P., Avenida de Brasília, 1449-006 Lisboa, Portugal; 2 MARETEC, Instituto Superior Técnico, Universidade de Lisboa, Av. Rovisco Pais, 1049-001 Lisboa, Portugal

## Abstract

**Background:**

Estuarine plumes are frequently under strong influence of land-derived inputs of organic matter. These plumes have characteristic physical and chemical conditions, and their morphology and extent in the coastal area depends strongly on physical conditions such as river discharge, tides and wind action. In this work we investigate the physical dynamics of the Tagus estuary plume and the CO_2_ system response during two contrasting hydrological winter periods. A hydrodynamic model was used to simulate the circulation regime of the study area, thus providing relevant information on hydrodynamic processes controlling the plume.

**Results:**

Model simulations show that for the studied periods, the major cause of the plume variability (size and shape) was the interaction between Tagus River discharge and wind. The freshwater intrusion on Tagus shelf exerted considerable influence on biochemical dynamics, allowing identification of two regions: a high nutrient region enriched in CO_2_ inside the estuarine plume and another warmer region rich in phytoplankton in the outer plume.

**Conclusions:**

The Tagus estuarine plume behaved as a weak source of CO_2_ to the atmosphere, with estimated fluxes of 3.5 ± 3.7 and 27.0 ± 3.8 mmol C m^−2^ d^−1^ for February 2004 and March 2001, respectively.

## Background

Coastal regions are significantly influenced by land-derived discharges emanating from estuaries, with estuarine plumes mediating the fluxes of natural terrestrial compounds and pollutant into shelf seas [1,2]. The extent and morphology of estuarine plumes are a direct consequence of river discharge, but are also strongly dependent on other physical conditions such as tide and wind stress.

An essential characteristic of estuarine plumes may be defined by a significant salinity gradient, although the boundary of the plume is often difficult to define given the highly dynamic nature of such systems [1]. Furthermore, highly stratified plumes lead to well defined density fronts along their boundaries, where turbidity is relatively low and chlorophyll *a* relatively high, even in winter [3].

Some studies concerning nutrients, fluxes of organic constituents and phytoplankton have been undertaken in estuaries and/or their associated plumes [1,4-8]; some highlight the seasonality CO_2_ source/sink behaviour of the estuarine plumes [9-12], and only a few refer the estuarine plume dynamics and the carbonate system response [13,14]. However, the CO_2_ uptake capacity of the estuarine plumes in several continental shelf zones is already extensively reported [2,3,15-20], suggesting that other estuarine plumes might counteract inner estuary CO_2_ emissions. The processes controlling the CO_2_ dynamics in the estuarine plume are linked to various factors such as spring/summer phytoplankton blooms, thermodynamic effects, winter floods from the inner estuary or stratification/mixing of the plume water column. On an annual basis, these processes together with the complexity of near shore ecosystems, can significantly impact water-air CO_2_ exchanges in estuarine plumes [13,14].

Significant drawdown of CO_2_ partial pressure (*p*CO_2_), biological uptake of dissolved inorganic carbon (DIC) and an associated enhancement of dissolved oxygen and pH within plumes occur due to enhanced biological activity, as reported for the Mississippi River plume in the USA [21,22], the Scheldt plume in Belgium [23], and the Pearl River estuary in China [8]. For the Changjiang Estuary plume (China), the *p*CO_2_ drawdown and DO enhancement in the warm seasons (from April to October) appeared to be controlled by primary productivity and water-air exchange, while mixing dominated the aqueous *p*CO_2_ in the cold seasons extending from November to March of the following year [15]. Mixing of river water with Gulf of Maine waters as also been pointed as responsible for the carbon variability in this system [24], although biological processes were significantly intense during the spring and summer seasons. Biological activity also lowers Amazon River plume *p*CO_2_, and contributes to a CO_2_ deficit in the northern western tropical North Atlantic Ocean that outlasts the plume’s physical structure [25].

**Figure 1:**
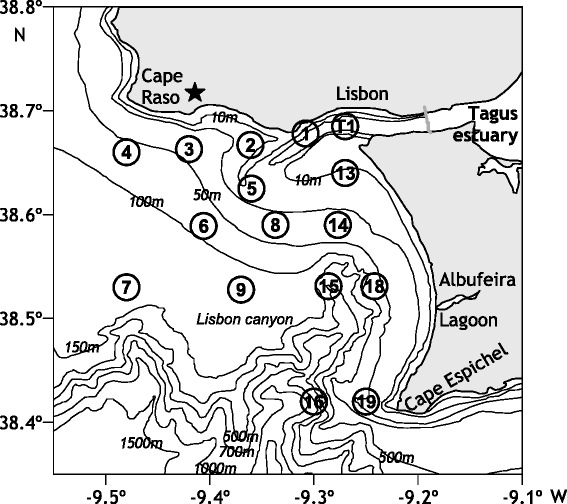
**Location of the study site.** Location of the sampling stations in the mouth of the Tagus estuary (SW Portugal) and adjacent coastal area. The position of the Guia meteorological station (38°41’27” N, 9°27’34” W) is marked with a star.

This paper aims to characterize the dynamics of water-air CO_2_ flux in the Tagus estuarine plume (Figure 1) during two contrasting winter periods, based on the *p*CO_2_ dynamics derived from field data. Underlying controlling mechanisms have been investigated based on the river discharge, the role of temperature and the biological activity. This study merges field data retrieved by experimental methods with information derived from the results of a numerical model on the spatial and temporal variability of the physical structure of the plume.

## Results and discussion

The sampling programs were carried out in winter, from 7 to 19 March 2001 and from 5 to 9 February 2004.

### Environmental settings

**Figure 2:**
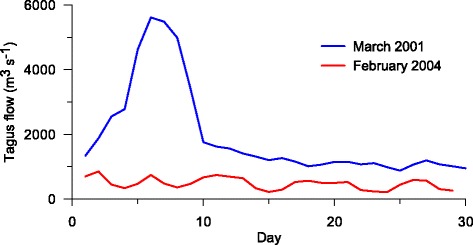
**Tagus river outflow.** Tagus river flow (m^3^ s^−1^) measured at a hydrometric station located upstream in March 2001 and February 2004. Values imposed in the modelled scenarios.

**Figure 3:**
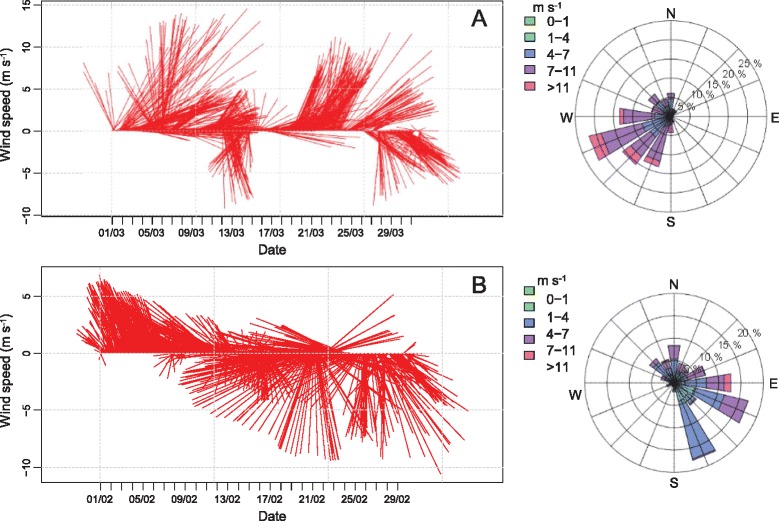
**Wind regime.** Stick diagram and wind rose of the wind regime measured at Guia meteorological station (38°41’27” N, 9°27’34” W) during **(A)** March 2001 and **(B)** February 2004. The wind intensity and direction showed here was used to force the model in each scenario. Wind rose shows the cumulative frequency in which wind speeds increase from the center to the outside.

The first three months of 2001, with mean air temperature of 13.0°C, were slightly warmer than the same period in 2004, with mean air temperature of 11.5°C. The winter 2001 was characterised by exceptional rain events, with precipitation values significantly higher than during the same period in 2004. The effect of the different rainy regimes is seen in the Tagus flow, with a mean value of 1893 m^3^s^−1^ in March 2001 and 481 m^3^ s^−1^ in February 2004 (Figure 2). Atmospheric CO_2_ (*p*CO_2,air_) was slightly lower (mean value of 373 μatm) in 2001, when compared with 2004 (mean value of 380 μatm). Both periods were characterised by absence of upwelling, seen in the positive Bakun index mean values of 725 m^3^ s^−1^ km^−1^ and of 344 m^3^ s^−1^ km^−1^ for March 2001 and February 2004, respectively. Significant shifts in wind direction and intensity were observed in March 2001 (Figure 3A), with dominant direction from the SW quadrant and intensities between 7 – 10 m s^−1^. In February 2004 the Tagus coastal area was under the influence of persistent south winds followed by stronger north winds (7 – 10 m s^−1^ in intensity), as shown in the wind rose in Figure 3B.

The winter periods were considered statistically different (t-test, *p* < 0.05, n = 27) for all physical (T, S) and biogeochemical parameters (Si(OH)_4_, AOU, SPM, Chl *a*, pH, TA, *p*CO_2_), except for DO, NO_3_, NH_4_ and PO_4_ (Table 1). Higher values for all parameters occurred in March 2001, except for S and pH, denoting the influence of the river plume. Salinity differences were also a consequence of the river flow in the two periods.

**Table 1:** **Mean values for monitored parameters**

	**MARCH 2001**		**FEBRUARY 2004**	
	**Range**	**Mean value (SD** ^**a**^ **)**	**Range**	**Mean value (SD** ^**b**^ **)**
*T* (°C)	14.8 – 15.9	15.4 (0.3)	14.4 – 14.9	14.7 (0.2)
*S*	31.4 – 34.6	32.3 (0.8)	27.9 – 35.7	32.7 (2.1)
NO_3_ (μmol l^−1^)	0.9 – 13.7	8.4 (3.5)	1.1 – 15.8	8.4 (5.9)
NH_4_ (μmol l^−1^)	0.5 – 3.3	1.9 (1.0)	0.4 – 4.4	2.6 (1.5)
PO_4_ (μmol l^−1^)	0.2 – 1.0	0.5 (0.2)	0.2 – 1.1	0.7 (0.3)
Si(OH)_4_ (μmol l^−1^)	4.8 – 26.5	17.1 (7.1)	1.1 – 16.9	9.6 (6.1)
DO (mg l^−1^)	8.3 – 8.9	8.5 (0.2)	7.6 – 9.3	8.2 (0.6)
AOU (μmol kg^−1^)	−25.9 – -1.7	−9.3 (6.8)	−35.6 – 25.8	2.0 (20.4)
SPM (mg l^−1^)	3.0 – 19.2	7.2 (4.6)	2.1 – 9.6	4.1 (2.2)
Chl *a* (mg m^−3^)	0.7 – 1.6	1.1 (0.3)	0.2 – 1.1	0.7 (0.3)
pH	7.88 – 7.96	7.93 (0.02)	8.06 – 8.17	8.10 (0.05)
TA (μmol kg^−1^)	3026 – 3770	3519 (282)	2357 – 2767	2489 (103)
*p*CO_2_ (μatm)	990 – 1467	1207 (140)	431 – 654	531 (70)
Wind speed (m s^−1^)	3.4 – 3.7	-	1.0 – 4.2	-
Piston velocity (cm h^−1^)	3.32 – 4.18	-	0.04 – 5.54	-

^a^standard deviation (SD) (n = 13).

^b^standard deviation (SD) (n = 14).

Seawater surface range of data and mean values for Tagus coastal area during winter 2001 and 2004. Shaded area indicates the parameters that are statistically different (t-test, *p* < 0.05, n = 27) between the 2001 and 2004 winter sampling periods.

TA values in March 2001 are considerably high, but fall within empirically established boundaries. They are within the range reported for Tagus estuary adjacent coastal waters in previous works [26,27]. Also, the Portuguese National Information System for Hydric Resources – SNIRH (data available at http://snirh.apambiente.pt/) reports TA values within the range 3000–8200 μmol kg^−1^ in the lower part of the estuary under the influence of freshwater. TA values in 2001 winter can be attributed to carbonate dissolution, which is confirmed by the significant decrease of particulate inorganic carbon from the estuary mouth (station T1, see Figure 1) to the plume. Although anaerobic degradation processes, such as denitrification and sulphate reduction, can also impact alkalinity increase, there are no evidences that such processes occurred during the March 2001 sampling period. Carbon loads to the plume were also quite different in both winter periods, being the value in the 2001 winter (2931 t C d^−1^) ~2.2 times higher than the value in 2004 winter (1340 t C d^−1^).

### The estuarine plume boundary

**Figure 4:**
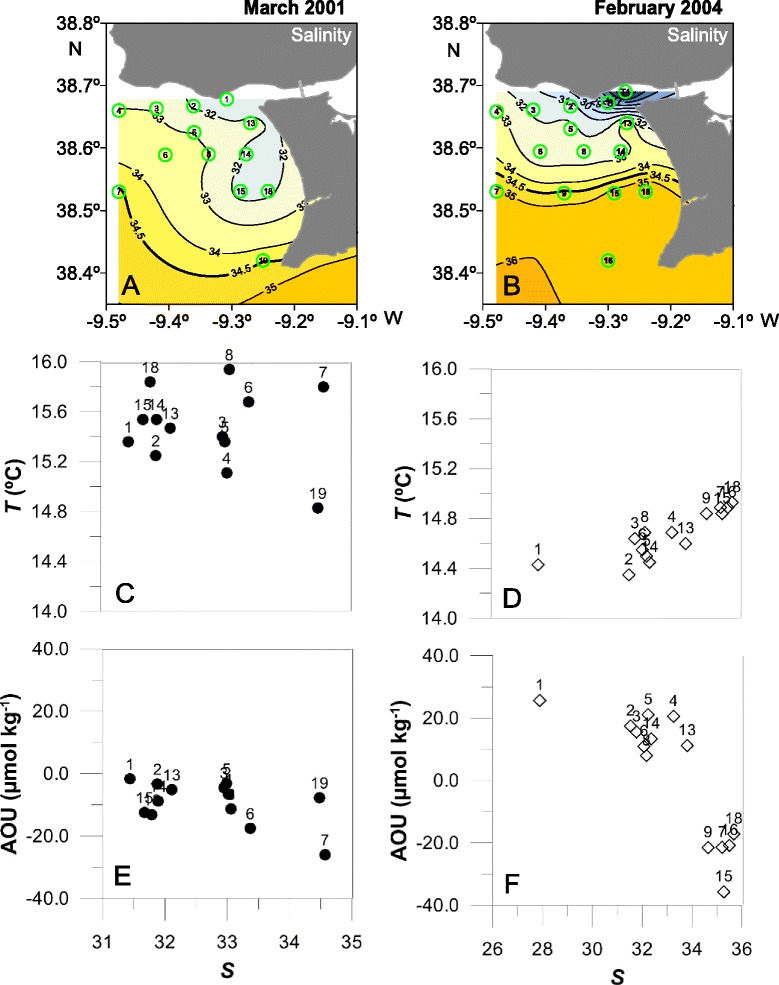
**Tagus plume characterization.** Surface salinity distribution during **(A)** March 2001 and **(B)** February 2004. T-S diagrams for **(C)** March 2001 and **(D)** February 2004 samplings, illustrating stations at the estuarine plume. AOU-S diagrams in **(E)** March 2001 and **(F)** February 2004 illustrating stations at the estuarine plume. The plume limit is represented by the 34.5 isopleth (bold line).

The boundary of Tagus plume can be inferred by the salinity gradient resulting from the fresh water intrusion in the coastal area. As such, the size and extension of the plume is strongly related with riverine discharges and, thus, with rainfall. This is observed in the studied periods (Figure 4). Using the salinity isopleth 34.5 to set up the limit of the plume, it is possible to notice a larger plume in March 2001 as a result of higher river flow. During this period the plume is more pronounced, extending south to Albufeira Lagoon reaching the Espichel Cape limit (Figure 4A), ~30 km from the estuary mouth. In February 2004, the plume remains closer to the Tagus mouth, extending ~14 km north-west along the coast (Figure 4B). The Τ-S (Figure 4C, D) and AOU-S (Figure 4E, F) diagrams reflect the impact of Tagus water input on the coastal area adjacent to the estuary in terms of salinity, temperature and oxygen. During March 2001 the Tagus Bay was under the influence of Tagus discharge, as noticed by the salinity values below 34.5, temperature higher than 15°C, and by the oxygen super-saturated water (AOU < 0) (Figure 4C, E).

**Figure 5:**
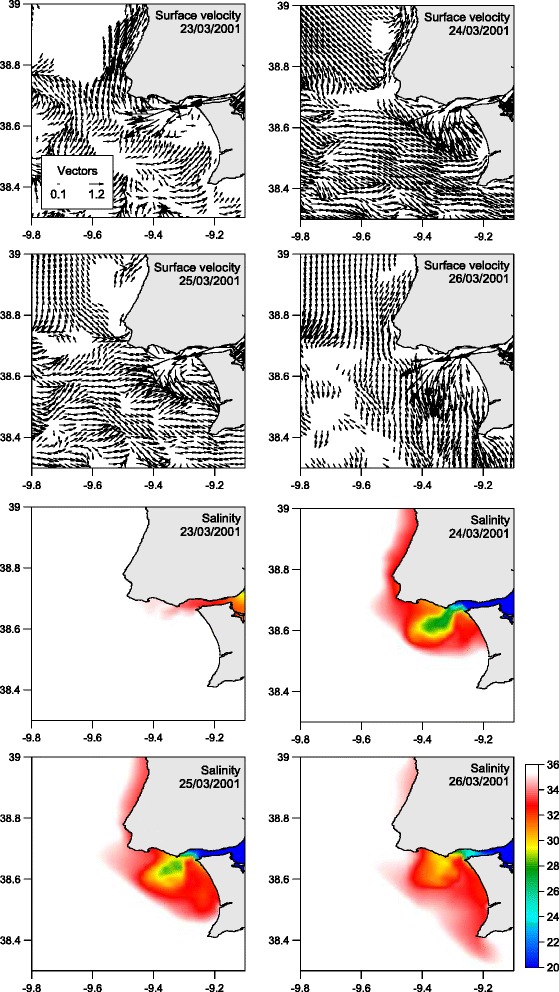
**Model results.** Model results for the surface currents and salinity in March 2001.

Model results show a significant variation in Tagus plume dispersion pattern between 20 and 30 March, as seen in Figure 5A and B. During this period, the horizontal current structure of the plume changes the northwest direction from the river mouth to the south. From 26 to 31 March the wind is consistently from the Northern quadrant with a relatively high intensity (~5 m s^−1^) (Figure 3), inducing marked offshore/southward advection of the estuary plume. Current velocity intensifies as a result of the river flow increase in this period. Model results for the salinity provide insights on the plume size and shape (Figure 5B), and show significant variation in its limits in response to the wind regime. It is also noticed an evolution from its original position trapped along the northern side of the river mouth, in 23 March, to a south-west transport off the Tagus mouth.

**Figure 6:**
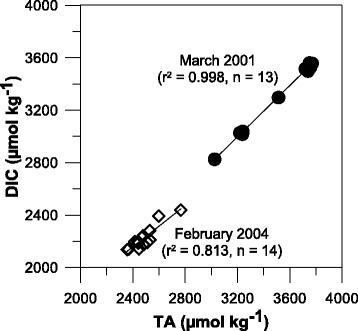
**DIC** ***vs*** **. TA.** Calculated dissolved inorganic carbon (DIC) *versus* total alkalinity (TA) in Tagus coastal area during March 2001 and February 2004.

The 2004 winter period was characterised by water temperatures below 15°C (Figure 4D). Oxygen saturation showed the presence of the undersaturated plume (Stations 1 to 6, 8, 13 and 14), and an outer oversaturated area (Stations 7, 9, 15, 16 and 18) (Figure 4F). This is reinforced by the calculated DIC *versus* TA plot (Figure 6), where the separation of the two water masses (riverine and oceanic) is observed. Station 1 (with S < 30) was clearly isolated from other stations near the northern coast, displaying salinities between 30 and 34.5 (Figure 4D, F).

**Figure 7:**
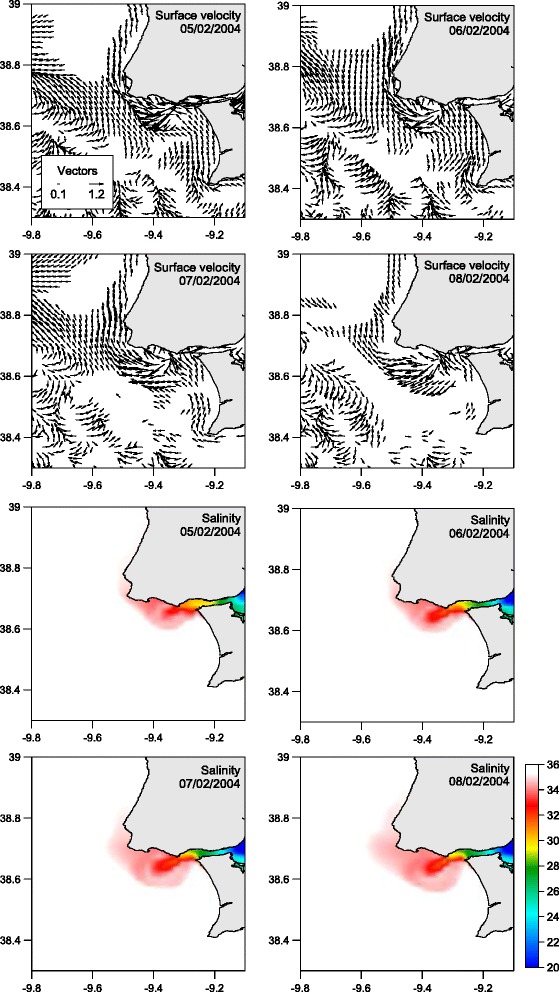
**Model results.** Model results for the surface currents and salinity in February 2004.

Simulated conditions for February 2004 show the formation of an estuary plume in the vicinity of the estuary mouth, extending westwards along the north side from the estuary as a result of geostrophic adjustment (Figure 7). This pattern is the result of the influence of moderate river flow and persistent south winds (Figure 3). Given the small variation in the forcing conditions, model results for February 2004 show little variation during the simulated period. The physical structure of the plume was consistently characterized by an offshore transport westward from the river mouth (Figure 7A), and presented a similar signature pattern in the horizontal salinity field observed in this period (Figure 7B).

**Figure 8:**
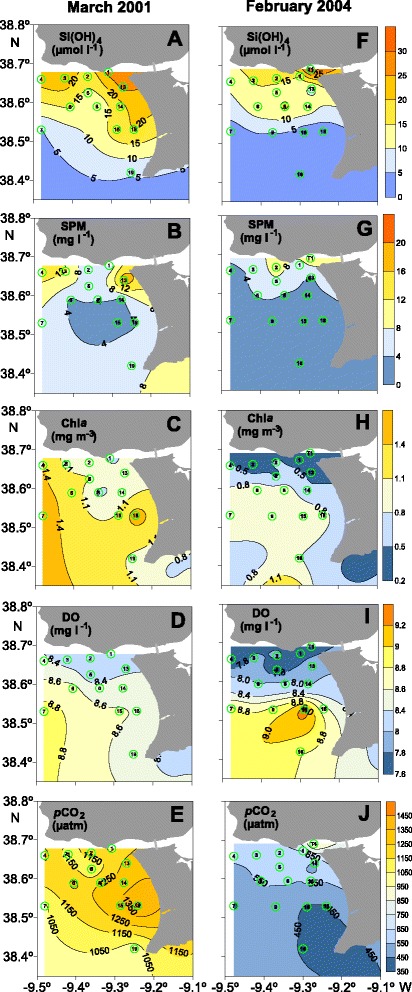
**Surface concentration of monitored properties.** Characterization of the Tagus coastal area during March 2001 and February 2004. Surface concentration of **(A,F)** silicate (Si(OH)4), **(B,G)** suspended matter (SPM), **(C,H)** chlorophyll *a* (Chl *a*), **(D,I)** dissolved oxygen (DO), and **(E,J)** calculated CO_2_ partial pressure (*p*CO_2_).

The signature of the estuarine plume is also evident in the chemical and biological parameters plotted in Figure 8. The results suggest a southward transport of the plume in winter 2001 and a northward transport in winter 2004. Model results explain these patterns by providing the temporal evolution of the physical conditions of the plume in both circumstances.

### The estuarine plume biogeochemistry

Contour plots show a marked estuarine plume enriched in nutrients, represented as Si(OH)_4_, particles and calculated *p*CO_2_ in 2001 (Figure 8A, B, E). In both sampling periods, high concentrations of suspended material (SPM) were associated with low concentrations of particulate organic carbon (data not shown), suggesting an organically impoverished plume, similar to other estuarine plumes [6,7,28]. SPM declined outside the plume, partly due to sinking, and there was a nutrient decrease caused by the combined effects of mixing with nutrient poor offshore waters and phytoplankton uptake, as suggested by the increase in Chl *a* (Figure 8C, H) and DO (Figure 8D, I).

The different water masses observed in both scenarios are characterized by distinct environmental properties, and also reveal particular CO_2_ features. Higher calculated *p*CO_2_ occur in March 2001 (Table 1), ranging from 990 μatm outside the plume (Station 19) to 1460 μatm near Albufeira Lagoon (Station 18) in the tip of the Lisbon submarine canyon head (Figure 8E). The elevated *p*CO_2_ values are probably a signal from the river where the most riverine station (~65 km of Station 1) values were up to 2500 μatm. In February 2004 the plume is pushed northwards (Figure 8J), and the highest value of calculated *p*CO_2_ (654 μatm) is observed at the estuary mouth (Station 1). Both periods were characterised by CO_2_ oversaturation, reaching ~400% in 2001 and ~170% in 2004. Other European estuarine plumes also presented CO_2_ oversaturation, such as the Scheldt in winter [9,12], the Elbe in the spring [6], and the Loire in autumn [5]. The marked *p*CO_2_ gradient in the buoyant plume suggests that its structure and dynamics regulates the *p*CO_2_ property in the studied area (Figure 8E, J). Again, several other studies revealed the variability of estuarine plumes with respect to CO_2_ dynamics [29].

**Figure 9:**
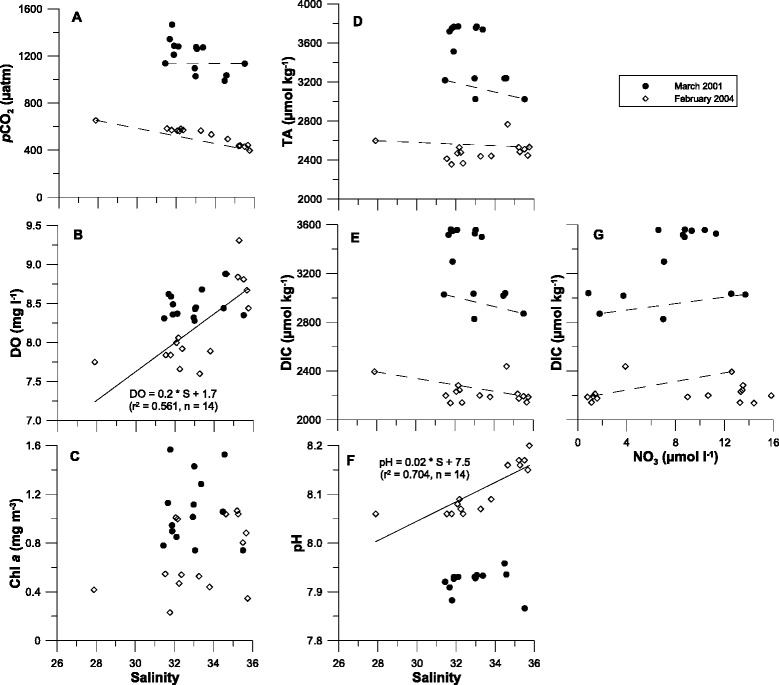
**Correlations between parameters.** Distributions of **(A)** calculated CO_2_ partial pressure (*p*CO_2_), **(B)** dissolved oxygen (DO), **(C)** chlorophyll *a* (Chl *a*), **(D)** total alkalinity (TA), **(E)** calculated dissolved inorganic carbon (DIC), and **(F)** pH along the salinity gradient, and of **(G)** DIC *versus* NO_3_ for Tagus coastal area during March 2001 and February 2004. End-member mixing line is represented by the dotted line.

Calculated *p*CO_2_ values decreased from inshore to offshore (Figure 8E, J), following the decreased in salinity. A significant correlation was found between calculated *p*CO_2_ and salinity in February 2004 (r^2^ = 0.890, *p* < 0.05, n = 14; Figure 9A). This distribution pattern, also seen in other systems [2,9,15,25], indicates that the mixing processes influence the CO_2_ pattern, which is reinforced by the proximity to the conservative mixing line (Figure 9A). However, the simultaneous calculated *p*CO_2_ and DO decrease (r^2^ = 0.561, *p* < 0.05, n = 14) and Chl *a* increases along the salinity gradient (Figure 9B, C), suggests the prominence of biological processes inside the plume. This is supported by the drawdown drop of calculated DIC associated with a pH increase (r^2^ = 0.704, *p* < 0.05, n = 14) (Figure 9E, F). The non-linear relationships between calculated DIC and nutrients (only represented by NO_3_ in this study) also reflect both mixing and biological processes (Figure 9G) regulating *p*CO_2_ distribution in February 2004. These features were also found in the Mississippi River [22] and Amazon River [25] plumes.

For the outer Loire estuary [13] biological processes, namely an episodic winter phytoplankton blooms, was also pointed out as responsible for *p*CO_2_ variability. Applying the Takahashi et al. [30] procedure to the February 2004 data, *p*CO_2_ variability was not affected by temperature. By contrast, in March 2001 *p*CO_2_ variability was attributed to physical processes, such as the thermodynamic effect of temperature and the riverine/estuarine discharge [31]. Moreover, the non-linear relationships of TA and DIC with salinity (Figure 9D, E) and of calculated DIC and NO_3_ (Figure 9G) reflect mixing and biological processes, inducing *p*CO_2_ variability. However, the calculated DIC *vs.* TA plot suggests that DIC and TA resulted by the same mechanism.

For the outer Loire estuary [13] biological processes, namely an episodic winter phytoplankton blooms, was also pointed out as responsible for *p*CO_2_ variability. Applying the Takahashi et al. [30] procedure to the February 2004 data, *p*CO_2_ variability was not affected by temperature. By contrast, in March 2001 *p*CO_2_ variability was attributed to physical processes, such as the thermodynamic effect of temperature and the riverine/estuarine discharge [31]. Moreover, the non-linear relationships of TA and DIC with salinity (Figure 9D, E) and of calculated DIC and NO_3_ (Figure 9G) reflect mixing and biological processes, inducing *p*CO_2_ variability. However, the calculated DIC *vs.* TA plot suggests that DIC and TA resulted by the same mechanism.

In both winter occasions two major regions were spatially individualized in the area: a high nutrient and CO_2_ enriched region inside the plume, and a warmer region characterized by higher phytoplankton biomass in the outer plume.

### CO_2_ fluxes across the water-air interface

The water-air CO_2_ fluxes showed similar patterns in the studied periods, with lower emissions to the atmosphere outside the plume (Table 2), and the highest values coincident with high wind speeds (data not shown). A striking pattern is found in both cases, namely the reduction in CO_2_ fluxes from inside the plume to outside of about 90% and 20%, in 2001 and 2004, respectively. Other authors [2,15-20] have also reported this CO_2_ uptake capacity of estuarine plumes, even suggesting that other estuarine plumes might counteract inner estuary CO_2_ emissions. Overall, the adjacent waters to the Tagus estuary acted as sources of CO_2_ to the atmosphere, emitting 25.9 ± 4.3 mmol C m^−2^ d^−1^ in March 2001, and 2.4 ± 3.4 mmol C m^−2^ d^−1^ in February 2004 (Table 2). Thus, CO_2_ emissions to the atmosphere in March 2001 were ~90% higher than in February 2004. The differences can be attributed to the variable river influence (e.g., effect of nutrients and labile organic matter, additional buoyant stability induced by freshwater fluxes), as suggested by other authors [10,25,32]. The CO_2_ emissions estimated in this work are within the range of those reported for the Tagus adjacent coastal waters [31] and several other near-shore ecosystems [9].

**Table 2:** **Water-air CO**_**2**_ **fluxes (Mean values and standard deviation)**

	**March 2001**	**February 2004**
Estuarine plume (S < 34.5)	27.0 ± 3.8	3.5 ± 3.7
Outer plume (S > 34.5)	19.9 ± 1.0	0.2 ± 0.1
Overall area	25.9 ± 4.3	2.4 ± 3.4

Mean values and standard deviation of water-air CO_2_ fluxes (mmol C m^−2^ d^−1^) calculated according to [32] parameterization for stations inside and outside the Tagus estuary plume during March 2001 and February 2004.

## Conclusions

Tagus estuarine plume can be traced on the shelf by gradients of salinity, but also by gradients of less conservative tracers such as water temperature, chlorophyll *a,* inorganic nutrients, total alkalinity and CO_2_. Thus, Tagus estuary adjacent shelf exhibits a high nutrient, low chlorophyll and enriched in CO_2_ estuarine plume, and a warm region impoverished in CO_2_ and enriched in phytoplankton in the outer plume. Estuarine Tagus plume behaved as a weak source of CO_2_ to the atmosphere, with estimated fluxes of 3.5 ± 3.7 and 27.0 ± 3.8 mmol C m^−2^ d^−1^ for February 2004 and March 2001, respectively.

Based on two winter cruises, it seems that Tagus plume significantly impacted estimates of water-air CO_2_ fluxes at a regional scale. Hence, this work emphasizes the importance of estuarine plumes on the CO_2_ dynamics in coastal areas. However, due to the complexity of near shore ecosystems and processes therein the magnitude of water-air fluxes is variable from one system to another.

Also, this study reinforces the usefulness of complimentary approaches such as the application of numeric models in reproducing the physical and chemical characteristics of plumes dynamics. The model results provide the temporal evolution of the plume under varying wind and rivers discharge, providing additional information that could not be obtained otherwise and, consequently, insightful clues on the integration of field data. Still, this is a first approach to using modelling tools with field data in the Tagus estuary, and future developments will include the CO_2_ dynamics in the model simulations.

## Methods

### Study area

The present investigation was carried out in the continental shelf offshore Tagus estuary (Figure 1) in the Portuguese coast, covering the geographic area between 38.35° – 38.80° N and 9.10° – 9.50° W. The continental shelf is ≤10 km wide south of Lisbon and presents topographic structures as prominent capes, promontories and submarine canyons. Its morphology is strongly influenced by the intense discharge of Tagus River, usually showing a pronounced dry/wet season signal as well as large inter-annual variation. The mean annual average discharge of Tagus is 350 m^3^ s^−1^ [33], with monthly averages ranging from 1 to 2200 m^3^ s^−1^. The Tagus estuary is a relatively shallow mesotidal system with semi-diurnal tidal regime (1 to 4 m in amplitude range). The surface area is about 320 km^2^ and the mean volume 1900×10^6^ m^3^. Intertidal mudflats cover an area of about 20 to 40% of the estuary.

The coastal area off Tagus estuary is characterized by the presence of upwelling plumes originated by jet-like flow extending more than 20 km seaward [34]. Advection of warmer oligotrophic oceanic waters into the shelf occurs during autumn and winter when southerly winds dominate, intensifying the poleward flow [35-37]. Episodes of reverse winds can occur during both seasons. In the absence of coastal upwelling, the surface circulation is predominantly northward [36] as a result of the geostrophic equilibrium. Also, the plume of estuarine waters is highly influenced by the coastline geometry. Intense freshwater discharge events under highly variable wind direction conditions in winter and strong upwelling episodes in spring-summer as well as fortnightly spring-neap tidal cycle, affect strongly the shape and size of Tagus plume [38]. While the plume is usually trapped close to the shore and transports estuarine water northward along the coast, under persistent northern wind conditions the plume is displaced offshore.

A significant amount of phytoplankton is exported from Tagus to the estuarine plume. Field and modelling studies suggest that nutrients are not depleted by primary producers due to light limitation inside the estuary and end up by being exported, eventually enhancing primary production in the coastal area [39-43]. Tagus estuary is also a major source of nutrients [43] and suspended matter to the adjacent coastal area [38]. The transport and transformation of such materials in the area is regulated by the interplay of dynamics and structure of the Tagus plume and hydrological characteristics of the coastal area [44].

The Tagus estuary and adjacent coastal waters are sources of CO_2_ to the atmosphere, with winter values ranging from 29 to 419 mmol C m^−2^ d^−1^ in the estuary, and up to 34 mmol C m^−2^ d^−1^ in the adjacent waters [45]. The carbonate system parameters have been evaluated in Tagus estuary and adjacent coastal area from 1999 to 2007 [46], and TA highest values (~4600 μmol kg^−1^) were recorded in 2002 spring [26].

### Sampling program

Surface water sampling was accomplished during ebb tide for a total of 16 stations distributed in the study area (see Figure 1), in two distinct winter periods (March 2001 and February 2004), defining winter as beginning in 21 December and ending in 21 March.

### Parameters determination

Temperature (T) and salinity (S; PSS-78) parameters were determined in situ with a Seabird SBE19/CTD (Conductivity - Temperature - Depth) probe. Salinity was calibrated with an AutoSal salinometer using IAPSO standard seawater, with a variation coefficient of 0.003%.

Dissolved oxygen (DO) was analysed following the Winkler method [47] using a whole-bottle manual titration, and the coefficient of variation associated with the method ranged from 0.08 to 0.25%. pH was measured immediately after sample collection at 25°C, using a Metrohm 704 pH-meter and a combination electrode (Metrohm) standardised against 2-amino-2-hydroxymethyl-1,3-propanediol seawater buffer (ionic strength of 0.7 M), at a precision of 0.005 pH units [48]. Total alkalinity (TA) samples were filtered through Whatman GF/F (0.7 μm) filters, fixed with HgCl_2_ and stored (refrigerated not frozen) until use. Samples were then titrated automatically with HCl (~0.25 M HCl in a solution of 0.45 M NaCl) past the endpoint of 4.5 [48], with an accuracy of ±2 μmol kg^−1^. The respectively accuracy was controlled against certified reference material supplied by A.G. Dickson (Scripps Institution of Oceanography, San Diego, USA). Discrete water samples were also taken for nutrient determination ($N{O}_3^{-}+N{O}_2^{-}$, referred as NO3; $N{H}_4^{+}$ referred as NH4; $P{O}_4^{3-}$, referred as PO4; $Si{(OH)}_4^{-}$, referred as Si(OH)4), chlorophyll *a* (Chl *a*) and suspended particulate matter (SPM). Nutrient samples were filtered through MSI Acetate Plus (0.45 μm) filters and analysed on a Traacs Autoanalyser, with a variation coefficient of ±1.0%. Chl *a* was measured by filtering triplicate aliquots of 250 ml water through Whatman GF/F (0.7 μm) filters under a 0.2 atm vacuum, which were immediately frozen and later extracted in 90% acetone for analysis in a fluorometer Hitachi F-7000, calibrated with commercial solutions of Chl *a* (Sigma Chemical Co.). The coefficient of variation associated with the method was 1.8%. For SPM measurements six aliquots of 750–1000 ml water samples were filtered through pre-combusted (2 h at 450°C) Wathman GF/F (0.7 μm) filters and determined gravimetrically (drying at 70°C). A portable Vaisala® meteorological station (Datalogger Campbell Scientific CR510) coupled with a MetOne 034A anemometer located at 11 m height was used to measure in situ wind speed and direction data at 1-minute intervals at each station. Wind speed was referenced to a height of 10 m (u_10_) using the algorithm given by Johnson [49]. We used one standard deviation of ±2 m s^−1^ as wind speed error.

### Calculated parameters

The upwelling indices (negative values indicate upwelling) were based on the northward wind stress component, and calculated according to Bakun [50]. Wind data was obtained from the meteorological weather station of Cape Carvoeiro located ~70 km north of Lisbon and supplied by the Portuguese Portuguese Institute for the Ocean and Atmosphere (IPMA, I.P.). Apparent oxygen utilisation (AOU) was calculated according to the equation:

1 $$ \mathrm{A}\mathrm{O}\mathrm{U} = {\mathrm{O}}_{2\mathrm{s}\mathrm{a}\mathrm{t}}\hbox{--}\ \mathrm{DO} $$

where O_2sat_ is the oxygen saturation in equilibrium with atmosphere. pH values corrected to in situ temperature were calculated from total alkalinity (TA) and in situ pH and temperature following the procedure proposed by Hunter [51]. For these calculations the carbon dioxide constants of Millero et al. [52] were applied. The partial pressure of CO_2_ in seawater (*p*CO_2_) and the dissolved inorganic carbon (DIC) were calculated from the in situ temperature, TA and corrected pH, using the carbonic acid dissociation constants given by Millero et al. [52] and the CO_2_ solubility coefficient of Weiss [53]. Errors associated with *p*CO_2_ and DIC calculations were estimated to be ±10 μatm and ±5 μmol kg^−1^, respectively (accumulated errors on TA and pH). The water-air CO_2_ fluxes (CO_2_ Flux) were computed according to the equation:

2 $$ \mathrm{C}{\mathrm{O}}_2\mathrm{Flux}=k.{K}_0.\Delta pC{O}_2 $$

where *k* is the gas transfer velocity (also referred to as piston velocity), *K*_*0*_ is the solubility coefficient of CO_2_ and Δ*p*CO_2_ the water-air gradient of *p*CO_2_. Positive fluxes indicate CO_2_ upward water – air emission. The *k* value is based on the Wanninkhof [54] parameterization. Atmospheric CO_2_ data were obtained from the Terceira Island’s reference station (Azores, Portugal, 38°46’N 27°23’W), operated by the network of the National Oceanic and Atmospheric Administration (NOAA)/Climate Monitoring and Diagnostics Laboratory/Carbon Cycle Greenhouse Gases Group [55]. Subsequently, the observed atmospheric CO_2_ content in mole fraction (in dry air) was converted into wet air values using the algorithms given by Dickson et al. [48]. Atmospheric *p*CO_2_ data obtained from our single day shipboard were only available for some sampling periods, while Terceira data represent a readily accessible continuous thropospheric dataset for the complete study period. Significant correlations were found between Terceira data and shipboard data available (r^2^ = 0.910, *p* < 0.05, n = 45). The discrepancies lie between 3 and 13 μatm, and the impact of using Terceira data on this study was considered negligible.

### Statistical analysis

Contour plots were created using Surfer 8.0® (Golden Software, 2002) following the kriging interpolation technique considering a linear interpolation with a slope of one. Exploratory analysis and statistical procedures were implemented using the statistical software Statistica 6.0® (Statsoft Inc., 2001). Differences between sampling periods in the measured/calculated physical-chemical and biological parameters were assessed using an analysis of variance (ANOVA), and differences between means have been considered statistically significant for *p* < 0.05.

### Model application

#### The model

The MOHID Water Modelling System (www.mohid.com) was applied to this study to simulate the circulation regime of the study area. MOHID is a three-dimensional marine model that has been implemented in several studies of estuaries and shelf circulation [56-60]. MOHID employs a 3D finite-volume approach for spatial discretization [61] using an Arakawa-C grid [62] to perform the computations. For the baroclinic force, the MOHID system uses a z-level approach with a partial step approach [63]. Temporal discretization is performed by a semi-implicit ADI (Alternating Direction Implicit) algorithm with two time levels per iteration. The hydrodynamic governing equations are the momentum and the continuity equations. The hydrodynamic model solves the primitive equations in Cartesian coordinates for incompressible flows.

The momentum and mass evolution equations are:

3 $$ \begin{array}{l}\frac{\partial {u}_i}{\partial t}+\frac{\partial \left({u}_i{u}_j\right)}{\partial {x}_j}=-\frac{1}{\rho_0}\frac{\partial {p}_{atm}}{\partial {x}_i}-g\frac{\rho \left(\eta \right)}{\rho_0}\frac{\partial \eta }{\partial {x}_i}\\ {}-\frac{g}{\rho_0}{\displaystyle \underset{x_3}{\overset{n}{\int }}\frac{\partial {p}^{\hbox{'}}}{\partial {x}_i}d{x}_3+\frac{\partial }{\partial {x}_j}\left(\upsilon \frac{\partial {u}_i}{\partial {x}_j}\right)-2{\varepsilon}_{ijk}{\varOmega}_j{u}_k}\end{array} $$

4 $$ \frac{\partial \eta }{\partial t}=-\frac{\partial }{\partial {x}_1}{\displaystyle \underset{-h}{\overset{\eta }{\int }}{u}_1d{x}_3}-\frac{\partial }{\partial {x}_2}{\displaystyle \underset{-h}{\overset{\eta }{\int }}{u}_2d{x}_3} $$

where *u*i is the velocity vector component in the Cartesian *x*i directions, η is the free surface elevation, ν is the turbulent viscosity and *p*atm is the atmospheric pressure. ρ’ is the density anomaly, ρ0 is the reference density, *g* is the acceleration of gravity, *t* is the time, *h* is the depth, Ω is the Earth’s velocity of rotation and ε is the alternate tensor.

The horizontal and vertical advection of momentum, heat and mass is computed using a Total Variation Diminishing (TVD) Superbee method [64]. Vertical turbulent viscosity/diffusivity coefficients are computed using a k-epsilon model coupling the MOHID system to the General Ocean Turbulence Model (GOTM) [65].

### Modelled scenarios

Two distinct winter episodes were modelled: March 2001 and February 2004. Both scenarios simulate oceanic conditions based on realistic forcing for river discharge (Figure 2) and wind conditions (Figure 3). We have adopted a method using a direct initialization with values from the MERCATOR solution [66]. This methodology interpolates the initial velocity field, temperature, salinity and sea surface height from the MERCATOR solution for the D2 grid assuming geostrophic balance. A two-month period was prescribed as a spin-up period.

### Model setup

The numerical model was implemented using a two level one-way nesting configuration. The first domain (D1) is a 2D barotropic tidal-driven model, forced only with the FES2004 (Finite Element Solution) tidal atlas [67,68]. This domain covers most of the Atlantic coast of Iberia and Northwest Morocco, and has variable horizontal resolution (0.02°-0.04°). The second (D2) level is a 3D baroclinic model with a 0.02° horizontal resolution and includes the Tagus Promontory area. This domain is directly coupled to D1 at the open boundaries using a one-way downscaling to impose the solution of D1. For D1 low-frequency open boundary conditions for salinity, temperature and U and V velocity components are interpolated via a downscaling of the MERCATOR operational solution for the Northeast Atlantic area (Mercator-Océan Psy2V3). A z-level vertical discretization was adopted for D2 with 33 vertical layers. In this application we have set a time step of 60 s for D1 and 15 s for D2.

Hourly values for wind, air temperature, relative humidity, barometric pressure and downward longwave and shortwave radiation, were used to calculate air-sea heat and momentum fluxes using bulk formulae. The data for atmospheric forcing was retrieved from an atmospheric modelling system based on the MM5 (Mesoscale Meteorological Model 5) model running at IST (http://meteo.ist.utl.pt). For land boundary conditions, the model uses realistic freshwater discharge and a null mass and momentum flux is imposed. River outflow was prescribed using outflow values from the Portuguese Water Institute (INAG) gauges for Tagus River.
